# Comparing the effectiveness of prostate cancer screening protocols: European Association of Urology– and European Randomized Study of Screening for Prostate Cancer–based strategies

**DOI:** 10.1002/ijc.70406

**Published:** 2026-02-25

**Authors:** Zhenwei Yang, Luuk A. van Duuren, Monique J. Roobol, Nicole S. Erler, Dimitris Rizopoulos, Eveline A. M. Heijnsdijk

**Affiliations:** ^1^ Department of Biostatistics Erasmus University Medical Center Rotterdam Rotterdam The Netherlands; ^2^ Department of Epidemiology Erasmus University Medical Center Rotterdam Rotterdam The Netherlands; ^3^ Department of Public Health Erasmus University Medical Center Rotterdam Rotterdam The Netherlands; ^4^ Department of Urology Erasmus University Medical Center Rotterdam Rotterdam The Netherlands; ^5^ Julius Center for Health Sciences and Primary Care University Medical Center Utrecht, Utrecht University Utrecht The Netherlands

**Keywords:** microsimulation, MRI triage, overdiagnosis, prostate cancer screening, risk‐adapted protocol

## Abstract

Prostate cancer (PC) screening reduces PC mortality but also causes burden such as overdiagnosis and unnecessary biopsies. The European Association of Urology (EAU) recently proposed a risk‐adapted screening protocol incorporating prostate‐specific antigen (PSA)‐based intervals, a risk calculator (RC), and magnetic resonance imaging (MRI), though its long‐term outcomes remain unquantified. We constructed the MIcrosimulation SCreening Analysis‐PSA model by adapting the existing MIcrosimulation SCreening Analysis‐PROstate microsimulation framework to simulate individual PSA trajectories. The model parameters were calibrated to outcomes of the European Randomized Study of Screening for Prostate Cancer (ERSPC). Using this model, we simulated five screening protocols for men aged 55–69: fixed 4‐year intervals between PSA tests and a biopsy when PSA ≥3.0 ng/mL (ERSPC protocol); PSA‐based intervals; MRI prior to biopsy; the RC and MRI prior to biopsy; and the full EAU protocol (PSA‐based intervals, RC, and MRI). Outcomes included PC mortality, overdiagnoses, the number of PSA tests, biopsies, and MRIs. Compared to the ERSPC protocol, PSA‐based intervals reduced PSA tests by 21%. The MRI‐only protocol decreased overdiagnosis by 6% but also required many MRIs. Incorporating the RC further reduced overdiagnosis to 10% and required 36% fewer MRIs than the MRI‐only protocol. The EAU combines the best of all these approaches while maintaining equal PC mortality (200 deaths per 10,000 men). The EAU protocol optimizes long‐term screening efficiency, significantly reducing biopsies and overdiagnosis with minimal mortality trade‐offs. MRI and RC integration enhance resource allocation.

## Graphical abstract

What's new?

Although screening reduces prostate cancer mortality, concerns about overdiagnosis and unnecessary biopsies have given rise to more tailored strategies. The European Association of Urology (EAU) proposed a risk‐adapted screening strategy combining prostate‐specific antigen (PSA)‐based intervals, a risk calculator, and magnetic resonance imaging (MRI). The present study evaluated the long‐term impact of this strategy using an adapted microsimulation screening analysis model. The model shows that PSA‐based intervals reduced PSA testing by more than one‐fifth, while the addition of a risk calculator further cut overdiagnosis and reduced MRI use. Overall, the EAU strategy preserves mortality benefits while reducing unnecessary testing and overdiagnosis.

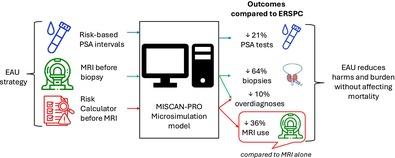

**Abbreviations**
EAU: European Association of Urology
ERSPC: European Randomized Study of Screening for Prostate Cancer
ISUP GG: International Society of Urological Pathology Grade Group
MISCAN‐PRO: MIcrosimulation SCreening Analysis‐PROstate
MRI: magnetic resonance imaging
PC: prostate cancer
PIRADS: Prostate Imaging Reporting and Data System
PLCO: Prostate, Lung, Colorectal and Ovarian Cancer Screening Trial
PRAISE‐U: PRostate cancer Awareness and Initiative for Screening Europe
PSA: prostate‐specific antigen
RC: risk calculator

## 1 INTRODUCTION

Prostate cancer (PC) screening is widely acknowledged to reduce PC mortality. The European Randomized Study of Screening for Prostate Cancer (ERSPC) showed a 20% mortality reduction using prostate‐specific antigen (PSA) screening.^1^ However, these screening programs incur considerable overdiagnosis,^2^ and they typically result in many unnecessary PSA tests and biopsies.^3^ There were ~750,000 unnecessary biopsies annually in the United States.^4^ To simplify decision‐making, most screening trials in the past (e.g., the ERSPC and the Prostate, Lung, Colorectal and Ovarian Cancer Screening Trial [PLCO]) used a fixed PSA threshold (usually 3.0 ng/mL) to distinguish abnormal cases for further investigation. Moreover, the test interval in screening programs is fixed, for example, four (two in the Swedish center) years^3^ in the ERSPC and 1 year^5^ in the PLCO trial.

Recently, there has been a noticeable increase in research advocating for more personalized screening programs to improve the balance between harms and benefits. Vickers et al.^6^ proposed the “traffic light” algorithm, assigning different screening intervals for different PSA levels. Furthermore, multiple studies^7^, ^8^, ^9^ have pointed out the promising benefits of implementing magnetic resonance imaging (MRI) in screening programs, which can target the biopsy in the suspicious area and reduce overdiagnosis.^10^ The European Association of Urology (EAU) released a new risk‐adapted algorithm in 2024, where a PSA test, a risk calculator (RC, considering PSA density), and MRI precede a biopsy. For men with PSA >3.0 ng/mL, first the RC (based on family history and general health information) is used to determine the need for an MRI. A biopsy follows if the MRI has positive results (Prostate Imaging Reporting and Data System [PIRADS] 3 or higher). Men with PSA between 1.0 and 3.0 ng/mL are exempted from further examinations and should repeat the PSA tests in 2–4 years. Men with PSA <1.0 ng/mL repeat the PSA test in 5 years if aged below 60 or stop screening if aged ≥60.^11^ This algorithm was implemented in the screening protocol of the ongoing PRostate cancer Awareness and Initiative for Screening Europe (PRAISE‐U) study.^12^ Although it is hypothesized to reduce the number of PSA tests and biopsies and mitigate overdiagnosis, the long‐term effects of this protocol are unknown.

This study aims to explore the long‐term clinical outcomes of the well‐known ERSPC screening protocol and the newly proposed EAU algorithm, in terms of outcomes such as overdiagnosis and PC mortality. To that end, we used and adapted the MIcrosimulation SCreening Analysis‐PROstate (MISCAN‐PRO) model.

## 2 METHODS

### 2.1 MISCAN‐PRO model

The MISCAN‐PRO model is a sophisticated microsimulation framework designed to map out the life trajectories of individuals in relation to PC.^13^ The model breaks down the PC natural history into 18 states, determined by tumor stages, Gleason scores, and metastasis. Individuals transition through these states using time‐to‐event modeling where an individual's duration in a state is sampled from probability distributions, reflecting the dynamic nature of the disease. These durations are determined using the same percentile within the dwell time distribution of each state, and therefore 100% correlated for an individual. The model also differentiates between two key phases: the pre‐clinical stage, where the disease is asymptomatic, and the clinical stage, beginning after detection by symptoms. Upon diagnosis, patients are directed to one of three primary management strategies: radiotherapy, radical prostatectomy, or active surveillance. In the absence of screening, the model assumes that individuals survive until PC‐related death, considering the patient's age and the characteristics of their tumor at diagnosis, or independently generated dates of other‐cause death, whichever comes first. This survival until PC‐related death is based on data from the pre‐PSA era (1983–1986) and adjusted to account for the significant improvements in survival rates that have occurred since then.^14^ Additionally, the model incorporates a hazard ratio of 0.65 to reflect the enhanced survival outcomes for non‐metastatic cases treated with radiotherapy or radical prostatectomy, underscoring the benefits of these interventions.

For a given screening strategy, the MISCAN‐PRO model simulates the life histories of a pseudo‐population from birth to death. Screening may alter these histories by detecting PC in an early stage, improving survival. Individuals may be cured due to screening, representing the mortality benefit. This is modeled as an extra probability that depends on the lead time (years by which cancer detection is advanced due to screening compared to the scenario without screening) and was implemented only for screen‐detected, non‐metastatic, and non‐overdiagnosed cases as cure probability = 1 – exp. (−cure parameter × lead time). Cured men will not die from PC. Men who are not cured die at the same time they would have died if they had not been screened. However, screening will also detect PCs that would never develop into cancer without screening, resulting in overdiagnosis. By simulating the same population under different screening protocols, the effect of these protocols can be quantified and compared.

After a positive PSA test, participants proceed with either biopsies or MRI where the simulated results of these tests depend on the corresponding sensitivity and specificity of the examination technique.

### 2.2 Model calibration

To mimic PSA‐based screening, we extended the MISCAN‐PRO model to simulate individual PSA values over time from a mixed‐effects model (named the MISCAN‐PSA model):

$$\log\left({\mathrm{PSA}}_{\mathrm{ij}}\right)=b_{0i}+b_{1}\frac{{\mathrm{age}}_{\mathrm{ij}}-40}{40}+\left(b_{2}+\frac{b_{6}}{\text{agecutoff}}\right)\max\left(0,\frac{{\mathrm{age}}_{\mathrm{ij}}-{\text{onsetage}}_{i}}{40}\right)+b_{3}I\left({\text{onset}}_{\mathrm{ij}}\right)r\_{\text{duration}}_{i}+b_{4}I\left(G6_{\mathrm{ij}}\right)+b_{5}I\left(G7{+}_{\mathrm{ij}}\right)+b_{6}f\left({\mathrm{age}}_{\mathrm{ij}},{\text{onsetage}}_{i},\text{agecutoff}\right)+\epsilon_{\mathrm{ij}},$$

wherefageijonsetageiagecutoff=0,ifonsetagei−ageij>agecutoff.agecutoff−onsetagei−ageijagecutoff,if0≤onsetagei−ageij≤agecutoff.1,ifonsetagei−agei<0.

${\mathrm{PSA}}_{\mathrm{ij}}$ and ${\mathrm{age}}_{\mathrm{ij}}$ are the PSA value and age for participant i at a time point j; agecutoff determines the number of years before cancer onset, which is defined as the first moment that cancer can be detected, an individual's PSA begins to increase at a fast rate; ${\text{onsetage}}_{i}$ is the individual's cancer onset age; $I\left({\text{onset}}_{\mathrm{ij}}\right)$ is the indicator function of PC onset, taking one if PC is present, and zero otherwise.; $r\_{\text{duration}}_{i}$ is the previously mentioned individual‐specific percentile between zero and one that determines the dwell time in each cancer state in MISCAN‐PRO and ensures the correlation of dwell times within an individual. As such, a negative b3 indicates PSA grows faster for individuals with a fast‐growing cancer; $I\left(G6_{\mathrm{ij}}\right)$ and $I\left(G7{+}_{\mathrm{ij}}\right)$ are indicator functions specifying whether the individual has a cancer with Gleason 6 and Gleason 7 or higher, respectively, allowing a jump in PSA after cancer onset and progression; $\epsilon_{\mathrm{ij}}\sim N\left(0,\sigma_{e}^{2}\right)$ is the residual term. The random intercept term follows a normal distribution, $b_{0i}\sim N\left(\mu_{0},\sigma_{0}^{2}\right)$, allowing individuals to have different baseline PSA.

The parameters varied in the calibration were: $\mu_{0}$, $\sigma_{0}$, *b*_1_–*b*_6_, $\sigma_{e}$ and agecutoff. To ensure the average PSA trajectory increases with time and disease progression, $b_{1}$, $b_{2}$, $b_{4}$, $b_{5}$, and $b_{6}$ were restricted to be positive, and $b_{5}>b_{4}$. $b_{3}$ was restricted to be negative, as individuals who progress faster through the different cancer stages should have higher PSA values. A list of the parameters to be calibrated is provided in Table S1.

All other parameters related to the natural history (i.e., onset probabilities, duration on each state, transition probabilities, etc.) were unchanged for this analysis. They have previously been calibrated to the ERSPC data stratified by age, year, and arm (screening vs. no screening).^14^ The assumed sensitivity and specificity of MRI, the combination of RC and MRI, biopsy alone, and the biopsy following the MRI are listed in Table 1.

**TABLE 1:** The sensitivity and specificity parameters for the magnetic resonance imaging (MRI), risk calculator (RC) + MRI and (MRI‐targeted and/or systematic) biopsies following the MRI.

	Sensitivity		Specificity
	Gleason 6	Gleason ≥7	‐
MRI	0.68^a^ (95% CI: 0.57–0.77)	0.89^a^ (95% CI: 0.82–0.94)	0.92^c^
RC + MRI	0.63^b^	0.85^b^	0.92^c^
MRI‐targeted and/or systematic biopsy	0.69^a^ (95% CI: 0.29–1.00)	0.92^a^ (95% CI: 0.79–1.00)	1.00
Systematic biopsy	0.55^a^ (95% CI: 0.25–0.83)	0.63^a^ (95% CI: 0.19–0.93)	1.00

Abbreviation: CI, confidence interval.

^a^ Based on the systematic review of Drost et al.^15^

^b^ Based on Drost et al.^15^ and Mannaerts et al.,^16^ see Supporting Information S1: supplementary methods.

^c^ Based on Hugosson et al.^17^

For calibration, we simulated a population of 1 million individuals with birth years, life expectancies and the screening policies, aligned with the ERSPC data across seven countries. The calibration targets were: (1) median PSA at age 40 of 0.6 ng/mL,^18^ (2) PSA distribution over four categories (<1.0, [1.0, 2.0], [2.0, 3.0], ≥3.0 ng/mL) in the first round of the ERSPC, (3) the proportion of PSA values >10 ng/mL in the first screening is <2%, (4) first‐round screening positive predictive value of a biopsy of 25%,^19^ (5) PSA distribution in the third‐round screening from the Rotterdam center of the ERSPC, stratified by the PSA in the first round, (6) PC diagnoses in the 16 years of follow‐up time from the ERSPC stratified by an individual's first‐round PSA value.^20^ The genetic algorithm^21^ and Nelder–Mead algorithm^22^ were used to optimize the parameters.

### 2.3 Screening scenarios

After calibration, we used the MISCAN‐PSA model to compare the effects of adding PSA‐based screening intervals, RC, and MRI to the ERSPC protocol. We simulated a cohort of 10 million men born in 1970 whose lifetimes follow the Dutch life table in 2019. We assumed 100% attendance for all steps, such as PSA test, RC, MRI, etc. Assumed sensitivities and specificities of the different test procedures were the same as in the calibration procedure (Table 1). Besides a scenario without PC screening, we investigated the following screening protocols:

*ERSPC protocol*:

Fixed 4‐year screening intervals (between age 55 and 70); biopsy after PSA >3.0 ng/mL.

*ERSPC‐PSA protocol*:

PSA‐based intervals (5 years if PSA <1.0 ng/mL and age <60, 4 years PSA ≥1.0 ng/mL, and stop screening if PSA <1.0 ng/mL and age ≥60); biopsy after PSA >3.0 ng/mL.

*ERSPC‐MRI protocol*:

After a PSA >3.0 ng/mL, individuals underwent an MRI and, if positive, an MRI‐guided and/or systematic biopsy.

*ERSPC‐RC‐MRI protocol*:

After a PSA >3.0 ng/mL, the RC determined eligibility for MRI. After a positive MRI, they did an MRI‐guided and/or systematic biopsy.

*EAU protocol*:

Combines PSA‐based intervals, RC, and MRI decisions.

In addition to the five above‐mentioned protocols, we investigated the following two protocols as sensitivity analyses:

*EAU‐variation protocol 1*:

The screening ends at the age of 75.

*EAU‐variation protocol 2*:

The screening interval for participants with PSA ≥1.0 ng/mL is 2 years.

The screening protocols were evaluated based on: PC mortality, screening and clinically detected cases by stage, number of PSA tests, MRIs and biopsies, overdiagnosis, and life years gained in the overall cohort. Overdiagnosis is defined as screening‐detected men who would not have been diagnosed during their lifetimes had there been no screening.

## 3 RESULTS

### 3.1 Model calibration

**FIGURE 1:**
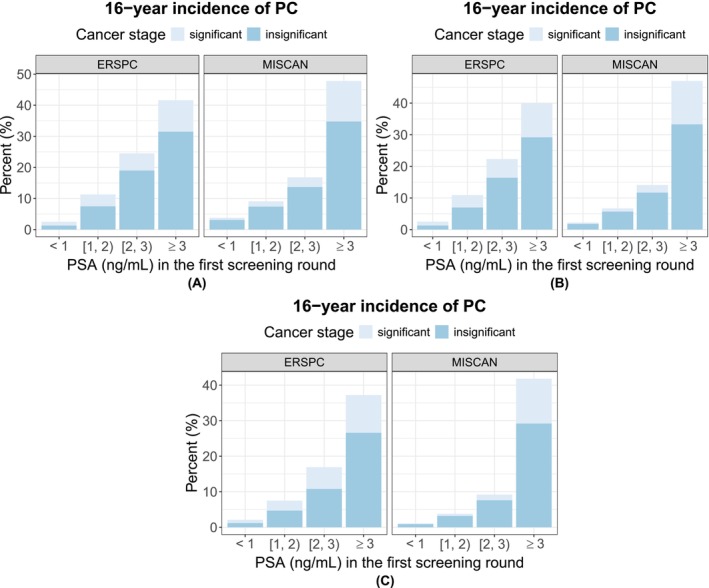
The 16‐year incidence of significant (Gleason 7 or higher cancer, i.e., International Society of Urological Pathology Grade Group [ISUP GG] 2 or higher) and insignificant (Gleason 6 cancer, i.e., ISUP GG 1) prostate cancer (PC), stratified by age and prostate‐specific antigen (PSA) result in the first screening round. Left panels show the incidence as observed in European Randomized Study of Screening for Prostate Cancer (ERSPC), right panels as predicted by MIcrosimulation SCreening Analysis (MISCAN)‐PSA. (A) Age 55–59. (B) Age 60–64. (C) Age 65–69.

**FIGURE 2:**
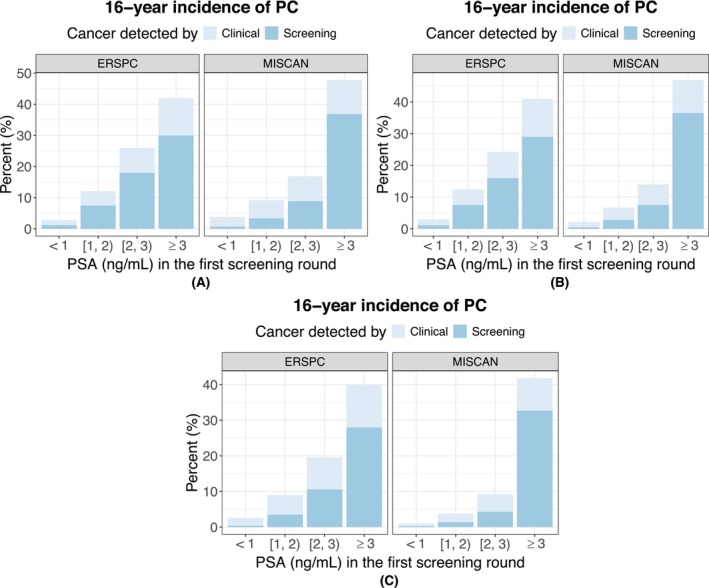
The 16‐year incidence of screening‐ and clinically detected prostate cancer (PC), stratified by age and prostate‐specific antigen (PSA) result in the first screening round. Left panels show the incidence as observed in European Randomized Study of Screening for Prostate Cancer (ERSPC), right panels as predicted by MIcrosimulation SCreening Analysis (MISCAN)‐PSA. (A) Age 55–59. (B) Age 60–64. (C) Age 65–69.

The calibrated MISCAN‐PSA model reproduced the PSA distributions in the first and third rounds of the ERSPC well (Figures S1 and S2). The incidence of significant (Gleason score of 7 or higher, i.e., ISUP 2 or higher) versus insignificant (Gleason score of 6, i.e., ISUP 1) PC stratified by PSA category and age at the first screening round is visualized in Figure 1. The corresponding incidence of clinical‐ versus screening‐detected PC is visualized in Figure 2. There was a clear pattern of increasing PC incidence with higher PSA values in the first round across all groups. The model generated similar incidence for people with PSA <1.0 ng/mL in both Figures 1 and 2. However, the model generally had lower incidence of significant PC in people whose first‐round PSA was between 1.0 and 3.0 ng/mL. The simulated incidence of screening‐detected PC in people with PSA between 1.0 and 3.0 ng/mL was lower than the corresponding observed incidence whereas that in people with PSA ≥3.0 ng/mL was higher.

Other calibration targets are presented in Table S2. We display 16‐year significant PC‐free survival by age and PSA level at the first screening in Figure S3.

### 3.2 Screening protocols

The outcomes for the five protocols are summarized in Table 2. Adapting the PSA‐based screening interval effectively reduced the number of PSA tests by around 22% compared to the ERSPC protocol (27,561 in the ERSPC‐PSA protocol and 27,648 in the EAU protocol vs. 35,089 in the ERSPC protocol for 10,000 men). Implementing MRI before further biopsies avoided around 64% of biopsies. Furthermore, adding the assessment of the RC further reduced the number of MRIs by around 36%. All other protocols except the ERSPC‐PSA protocol detected more significant PC and fewer insignificant PC during the screening and thus lowered overdiagnoses by 6%–10%. The PC mortality of all protocols was comparable to that of the ERSPC protocol, with relative differences ranging from −3% to 0%. Implementing MRI before biopsies even decreased the PC mortality by 2% and increased the life years gained by 5%.

**TABLE 2:** The clinical outcomes of the no‐screening scenario and the five protocols based on the European Randomized Study of Screening for Prostate Cancer (ERSPC) and European Association of Urology (EAU) screening protocol (per 10,000 men, relative differences to the ERSPC protocol in parentheses).

	No screening	ERSPC	ERSPC‐PSA	ERSPC‐MRI	ERSPC‐RC‐MRI	EAU
PSA tests	0	35,089	27,561 (−22%)	35,131 (+0%)	35,175 (+0%)	27,648 (−21%)
MRIs	0	0	0	4152	2642	2631
Screening biopsies	0	4103	4090 (−0%)	1483 (−64%)	1427 (−65%)	1422 (−65%)
Clin insig PC	683	478	480 (+0%)	502 (+5%)	512 (+7%)	514 (+8%)
Clin sig PC	727	361	362 (+0%)	345 (−5%)	355 (−2%)	356 (−1%)
Screen insig PC	0	779	774 (−1%)	690 (−11%)	649 (−17%)	646 (−17%)
Screen sig PC	0	217	218 (+1%)	271 (+25%)	273 (+26%)	276 (+27%)
Total PC	1410	1835	1834 (−0%)	1808 (−1%)	1789 (−3%)	1792 (−2%)
Overdiagnoses	‐	425	425 (+0%)	397 (−6%)	381 (−10%)	382 (−10%)
PC deaths	331	199	200 (+1%)	195 (−2%)	200 (+1%)	200 (+1%)
Life years gained	‐	1273	1261 (−0%)	1332 (+5%)	1289 (+1%)	1281 (+1%)

*Note*: Life years gained were calculated with reference to the life years in the no screening scenario.

Abbreviations: Clin insig, clinical insignificant; Clin sig, clinical significant; insignificant PC, prostate cancer with Gleason score of 6; MRI, magnetic resonance imaging; PC, prostate cancer; PSA, prostate‐specific antigen; Screen insig, screening insignificant; Screen sig, screening significant; significant PC, prostate cancer with Gleason score of 7 or higher.

Extending the maximum screening age to 75 resulted in 13% lower PC mortality than the EAU protocol (Table 3). However, this protocol overdiagnosed the most people across all scenarios, 60% more than the EAU protocol and 44% more than the ERSPC protocol. The number of PSA tests increased by 18% whereas the number of biopsies and MRIs was almost 53% higher compared to the EAU protocol. Similarly, shortening the PSA screening interval from 4 to 2 years reduced PC mortality by 16% relative to the EAU protocol. As a trade‐off, the EAU‐variation protocol 2 caused 39% more overdiagnoses than the EAU protocol. Halving the screening interval also almost doubled the number of MRIs, resulting in around 50% more PSA tests and biopsies.

**TABLE 3:** The clinical outcomes of the two variations of the European Association of Urology (EAU) protocol with reference to the EAU protocol and no‐screening scenario (per 10,000 men, relative differences relative to the EAU protocol in the round brackets).

	No screening	EAU	EAU‐variation 1: end age 75	EAU‐variation 2: interval 2 years
PSA tests	0	27,648	32,576 (+18%)	40,138 (+45%)
MRIs	0	2631	4027 (+53%)	4658 (+77%)
Screening biopsies	0	1422	2066 (+45%)	2085 (+46%)
Clin insig PC	683	514	451 (−12%)	427 (−17%)
Clin sig PC	727	356	252 (−29%)	275 (−23%)
Screen insig PC	0	646	913 (+41%)	978 (+51%)
Screen sig PC	0	276	404 (+46%)	261 (−6%)
Total PC	1410	1792	2020 (+13%)	1941 (+8%)
Overdiagnoses	‐	382	611 (+60%)	531 (+39%)
PC death	331	200	173 (−13%)	168 (−16%)
Life years gained	‐	1281	1439 (+12%)	1575 (+23%)

*Note*: Gained life years were calculated with reference to the life years in the no‐screening scenario.

Abbreviations: Clin insig, clinical insignificant; Clin sig, clinical significant; insignificant PC, prostate cancer with Gleason score of 6; MRI, magnetic resonance imaging; PC, prostate cancer; PSA, prostate‐specific antigen; Screen insig, screening insignificant; Screen sig, screening significant; significant PC, prostate cancer with Gleason score of 7 or higher.

## 4 DISCUSSION

This study extends the MISCAN‐PRO model with a mixed‐effect model to simulate the PSA measurements for participants over time. This extended MISCAN‐PSA model evaluated five screening programs based on the recently proposed EAU protocol and the well‐known ERSPC protocol. Compared to the ERSPC protocol, the EAU protocol, combining PSA‐based intervals, MRI, and RC, enhances screening efficiency without increasing mortality. By disentangling the effects of each component, we found that (1) PSA‐based intervals are the main driver behind the 21% reduction in PSA tests; (2) MRI before biopsy reduces the number of biopsies by 64%, improves detection of significant cancers, and lowers overdiagnosis by 6%; and (3) introducing the RC before MRI further reduces MRI use by 36%. The sensitivity analyses demonstrated that extending screening or shortening the screening intervals further decreased PC mortality, but markedly raised overdiagnosis.

This is the first study that utilizes an established simulation model to evaluate the newly proposed EAU protocol and directly compare it with the well‐known ERSPC protocol. The MISCAN‐PRO model used in this study is a widely recognized and reliable modeling framework that enables the investigation of long‐term outcomes associated with different PC screening strategies and supports both large‐scale policy development and individualized patient care. The natural history of the model was previously calibrated on one of the most extensive datasets with long‐term follow‐up: the ERSPC data.^14^ The MISCAN‐PSA model, inheriting the natural history‐related framework from the MISCAN‐PRO model, is also equipped with the functionality to generate subject‐specific PSA trajectories. It is a strength that both the natural history component and the PSA module are calibrated to the data from the same randomized controlled trial. Using microsimulation, risk‐adaptive screening programs can be evaluated more thoroughly based on long‐term outcomes (e.g., mortality) that are hard to observe due to the expense in practice. Furthermore, other causal factors, for example, overdiagnosis, can be easily calculated by comparing the population under screening and the exact same population in the counterfactual world without screening.

Although the model provides useful insight into the research question, its conclusions should be interpreted with appropriate caution given the data limitations. First, in calibrating the PSA model, only two rounds of PSA distributions were used instead of individual PSA data, which constrained its flexibility. Thus, only the random intercept was considered in the PSA model, whereas the random slopes were not assumed. As a compromise, we added a slope change term 13 years before PC onset to allow a more biologically correct PSA trajectory development. The PSA model might be improved by incorporating additional nonlinear trajectories via splines for both the fixed and random effects of the PSA model.^23^, ^24^ Second, in the calibration phase, the MISCAN‐PSA model tended to generate more screen‐detected cases for people with PSA >3.0 ng/mL in the first round. This indicates that people who will develop cancer are more likely to have PSA >3.0 ng/mL already in the first round than to have PSA between 1.0 and 3.0 ng/mL in the model. In this case, using MRI and RC, more patients with significant PC will be detected in the model. Third, our calibration focused on the commonly used definition of significant cancer as ISUP GG2 or higher and the screening ages 55–69. Our results will change when using ISUP GG 3 or higher as the definition of significant disease or screening other age groups. Finally, the test characteristics in the model were assumed to be constant for simplicity, which, in real practice, can vary across different screening rounds. Moreover, the test characteristics used in the MISCAN‐PSA model were extracted from previous literature while their corresponding uncertainty was not considered. Some of these sensitivities and specificities, such as those of MRI, were difficult to obtain because of the different populations in the study. Given these limitations, some uncertainty in long‐term projections is unavoidable. However, the model is still suitable for comparing relative differences between screening strategies under consistent assumptions, as was done in this study.

This study evaluated commonly used PC screening protocols applied to a 55‐ to 69‐year‐old population by enhancing an established microsimulation model. Using a PSA‐based screening interval strategy mainly reduces the number of PSA tests. Using MRI before biopsies helps decrease overdiagnosis and the number of biopsies. Using an RC to determine who receives MRI testing reduces the number of MRIs. The combination of all these strategies, as proposed in the recent EAU protocol, results in all the benefits mentioned above while having no substantial effect on PC mortality.

## AUTHOR CONTRIBUTIONS

**Zhenwei Yang:** Methodology; software; formal analysis; validation; investigation; writing – review and editing; writing – original draft; visualization. **Luuk A. van Duuren:** Software; validation; investigation; writing – review and editing. **Monique J. Roobol:** Resources; writing – review and editing. **Nicole S. Erler:** Methodology; writing – review and editing; supervision. **Dimitris Rizopoulos:** Supervision; methodology; writing – review and editing. **Eveline A. M. Heijnsdijk:** Conceptualization; methodology; validation; investigation; writing – review and editing; supervision.

## FUNDING INFORMATION

The research was funded by the National Institutes of Health (the NIH CISNET Prostate Award CA253910).

## CONFLICT OF INTEREST STATEMENT

The authors declare no potential conflict of interest.

## Supporting information


**Data S1.** Supporting Information.

## Data Availability

Aggregated data, from Remmers et al.,^20^ used as input for the MISCAN‐PSA or MISCAN‐PRO model can be requested from the primary source via e.heijnsdijk@erasmusmc.nl. The model outcomes that support the findings of this study are available from the corresponding author upon reasonable request.
