# Supplementary material for: Comparing the effectiveness of prostate cancer screening protocols: European Association of Urology– and European Randomized Study of Screening for Prostate Cancer–based strategies
Source: Int J Cancer. 2026 Feb 25;159(2):432–9. doi: 10.1002/ijc.70406 (PMC13193407; doi:10.1002/ijc.70406)
Supplement: Supplementary file 1 — Data S1. Supporting Information. [file IJC-159-432-s001.pdf]

Supplement Material - Comparing the Effectiveness of Prostate Cancer Screening Protocols: European Association of Urology- and European Randomized Study of Screening for Prostate Cancer-based Strategies

Zhenwei Yang, Luuk A. van Duuren, Monique J. Roobol, Nicole S. Erler, Dimitris Rizopoulos,

Eveline A.M. Heijnsdijk

Table of Contents

Supplementary methods **2**

Sensitivities of the combination of MRI and the risk calculator (RC) **2**

Supplementary results **3**

Calibrated parameters **3**

Table S1: List of calibrated parameters in the mixed model to simulate PSA trajectories 3

Parameter Targets **4**

Figure S1: The PSA distributions of the first screening round as predicted by the MISCAN and observed in ERSPC across three age groups (55 to 59 years old, 60 to 64 years old, and 65 to 69 years old). 4

Figure S2: The PSA distributions of the third screening round as predicted by the MISCAN and observed in ERSPC, stratified by the individual's PSA value in the first round (< 1.0 ng/mL, 1.0 to 3.0 ng/mL, and > 3.0 ng/mL). 5

Table S2: results of other calibration targets 6

Figure S3: The significant (Gleason 7 or higher cancer) prostate cancer (PC)-free survival in 16 years after the first screening round (upper panel) and the number of people under risk at 0, 4, 8, 12 and 16 years after first screening round (lower panel), stratified by age: (A) 55-59, (B) 60-64, and (C) 65-69 years, and PSA result in the first screening round. 8

Reference **9**

Supplementary methods

Sensitivities of the combination of MRI and the risk calculator (RC)

The sensitivities of RC+MRI presented in Table 1 of the manuscript were derived from a synthesis of findings by Drost et al.[1] and Mannaerts et al.[2]. As Drost et al.[1] have noted, the sensitivity of MRI for Gleason 6 patients is 0.68, and for Gleason > 7 patients, it is 0.89.

Based on Figure 1 in Mannaerts et al.[2], in the absence of a risk calculator, 51 individuals (49 + 2) with Gleason >7 would be detected, in which two people were missed by the risk calculator. Therefore, the sensitivity of RC+MRI (for Gleason > 7 patients) should be adjusted downward by approximately $\frac{2}{2+49} \approx 4\%$. This yields a combined sensitivity of:

RC+MRI (Gleason >7) $= 0.89 \times 96\% = 0.85.$

Likewise, for Gleason 6 patients, 14 individuals (13 + 1) would be detected without the risk calculator, with one missed due to its use. This corresponds to a reduction of approximately $\frac{1}{1+13} \approx 7\%$, resulting in:

RC+MRI (Gleason 6) $= 0.68 \times 93\% = 0.63.$

Supplementary results

Calibrated parameters

Table S1: List of calibrated parameters in the mixed model to simulate PSA trajectories

Parameter	Initial value	Range	Final value	Note
μ_0	-0.54	(-1, 0.1)	-0.24	Fixed effect of the intercept, i.e., the mean PSA at age 40 is exp(-0.24)
σ_0	0.36	(0.05, 1)	0.49	
b_1	0.01	(0, 3)	1.02	Fixed effect of age on PSA
b_2	0.5	(0, 3)	0.00	
b_3	-0.5	(-3, 0)	-0.02	Fixed effect of duration
b_4	0.5	(0, 3)	1.03	Log PSA growth at Gleason 6
b_5	0.6	(0, 3)	1.03	Log PSA growth at Gleason 7 or 8, should be at least larger than b_4
b_6	0.5	(0, 3)	0.93	Average PSA growth before PSA onset
σ_e	0.61	(0.05, 1)	3.05	Residual standard deviation
agecutoff	25	(1, 30)	13.10	Years before cancer onset that triggers the increase of PSA

Parameter Targets

1. PSA distribution in first screening round

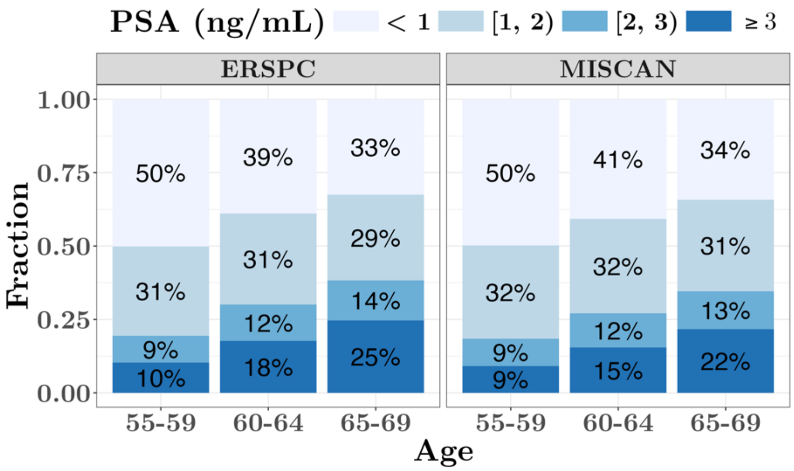

Figure S1: The PSA distributions of the first screening round as predicted by the MISCAN and observed in ERSPC across three age groups (55 to 59 years old, 60 to 64 years old, and 65 to 69 years old).

2. PSA distribution in the third screening round

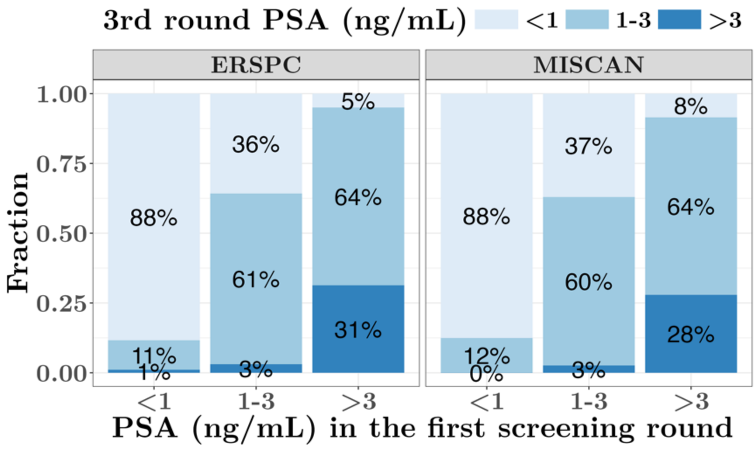

Figure S2: The PSA distributions of the third screening round as predicted by the MISCAN and observed in ERSPC, stratified by the individual's PSA value in the first round (< 1.0 ng/mL, 1.0 to 3.0 ng/mL, and > 3.0 ng/mL).

3. Other calibration target

Table S2: results of other calibration targets

	Target	MISCAN final output
**Median PSA at age 40 (ng/mL)**	0.60	0.60
**Proportion of PSA values > 10 ng/mL in the first screening round**	0.02	0.06
**Proportion of positive biopsies in the first screening round**	0.25	0.24

4. **Significant cancer-free survival within 16 years after the first screening round in 1993 from the MISCAN simulated data**

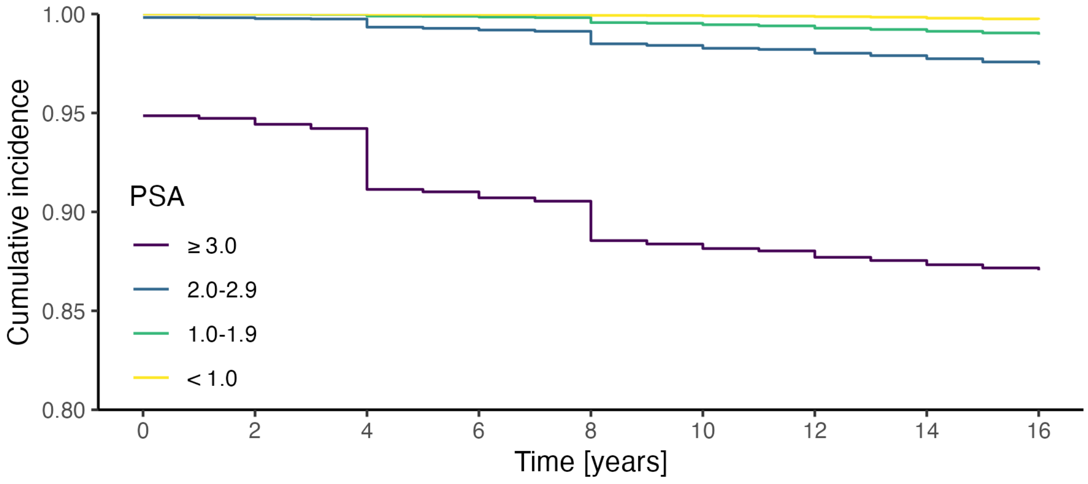

Number at risk

PSA level					
< 1.0	16794	15872	14813	13336	11379
1.0-1.9	10703	10102	9396	8446	7193
2.0-2.9	3134	2942	2715	2434	2068
≥ 3.0	3080	2657	2408	2161	1865

(A) 55-59

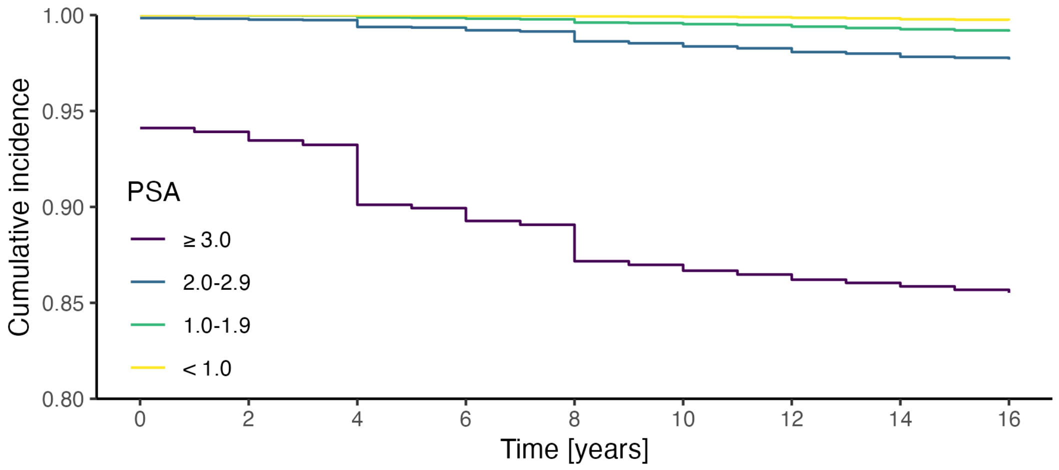

Number at risk

PSA level					
< 1.0	8926	8137	7238	6063	4636
1.0-1.9	7062	6426	5696	4748	3601
2.0-2.9	2552	2313	2040	1697	1288
≥ 3.0	3376	2787	2412	2028	1580

(B) 60-64

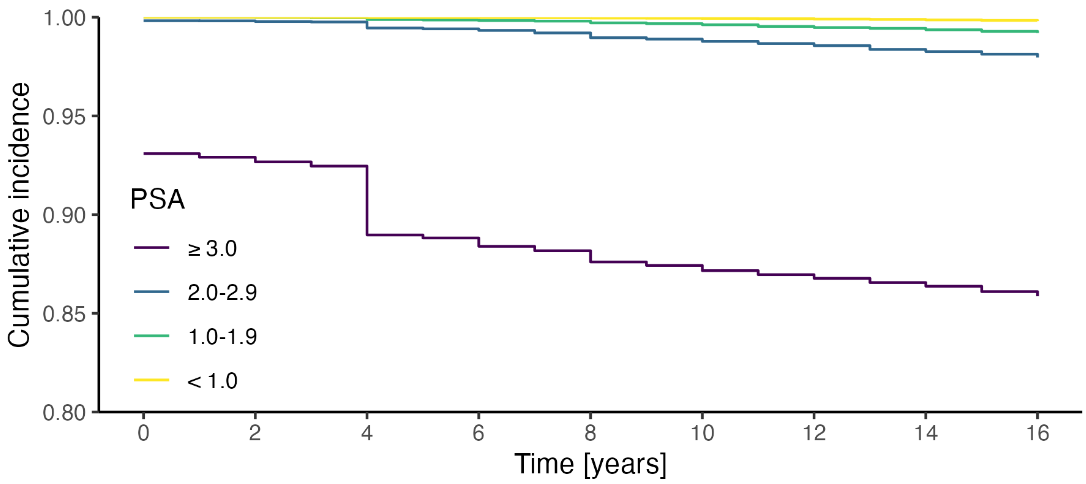

Number at risk

PSA level					
< 1.0	5906	5061	4158	3093	1990
1.0-1.9	5381	4597	3776	2811	1799
2.0-2.9	2236	1912	1574	1183	756
≥ 3.0	3739	2862	2354	1806	1242

(C) 65-69

Figure S3: The significant (Gleason 7 or higher cancer) prostate cancer (PC)-free survival in 16 years after the first screening round (upper panel) and the number of people under risk at 0, 4, 8, 12 and 16 years after first screening round (lower panel), stratified by age: (A) 55-59, (B) 60-64, and (C) 65-69 years, and PSA result in the first screening round.

Reference

1. Drost F-JH, Osses D, Nieboer D, et al. Prostate Magnetic Resonance Imaging, with or Without Magnetic Resonance Imaging-targeted Biopsy, and Systematic Biopsy for Detecting Prostate Cancer: A Cochrane Systematic Review and Meta-analysis. *European Urology*. 2020/01/01/ 2020;77(1):78-94. doi:https://doi.org/10.1016/j.eururo.2019.06.023

2. Mannaerts CK, Gayet M, Verbeek JF, et al. Prostate Cancer Risk Assessment in Biopsy-naïve Patients: The Rotterdam Prostate Cancer Risk Calculator in Multiparametric Magnetic Resonance Imaging-Transrectal Ultrasound (TRUS) Fusion Biopsy and Systematic TRUS Biopsy. *European Urology Oncology*. 2018/06/01/ 2018;1(2):109-117. doi:https://doi.org/10.1016/j.euo.2018.02.010
